# Effects of Dietary *Bacillus* spp. and *Bacillus* spp.-Fermented Soybean Hull on Growth Performance and Apparent Nutrient Digestibility in Growing–Finishing Pigs

**DOI:** 10.3390/ani16203224

**Published:** 2026-10-14

**Authors:** Jingxin Cao, Shuaihu Chen, Jialin Li, Wenju Zhang, Xihong Zhou

**Affiliations:** 1 College of Animal Science and Technology, Shihezi University, Shihezi 832000, China; 2 Institute of Subtropical Agriculture, Chinese Academy of Sciences, Changsha 410125, China; 3 College of Veterinary Medicine, Hunan Agricultural University, Changsha 410128, China; 4 Veterinary Medicine and Engineering, Nanyang Vocational College of Agriculture, Nanyang 473061, China; 5 Bingtuan Key Laboratory for Efficient Utilization of Non-Grain Feed Resources, Shihezi University, Shihezi 832000, China

**Keywords:** *Bacillus*, fermented feed, finishing pigs, growth performance, soybean hull

## Abstract

This study investigated the fermentation improvement of soybean hull by *Bacillus subtilis* and *Bacillus licheniformis* from Ningxiang pigs and evaluated different feeding methods in growing-finishing pigs. The cellulase-producing capacity of the two strains and their fermentation effects on soybean hull were determined. Compound *Bacillus* preparation (200 and 400 g/t) and fermented soybean hull (5% and 10%) were supplemented into finishing pig diets, and their effects on growth performance; apparent nutrient digestibility; and serum biochemical, immune, and antioxidant indices were evaluated. In vitro results showed that combined fermentation significantly decreased crude fiber content and increased crude protein and ether extract concentrations. Animal trial results showed that supplementation with 400 g/t compound *Bacillus* preparation or 5% fermented soybean hull significantly increased final body weight but did not significantly affect average daily gain (ADG), average daily feed intake (ADFI), or feed-to-gain ratio (F/G). In the compound *Bacillus* preparation trial, supplementation increased the apparent digestibility of crude protein and gross energy and was associated with changes in several serum antioxidant and inflammatory indicators. In the fermented soybean hull trial, supplementation improved crude protein utilization efficiency, with no significant effects on the measured serum immune parameters. Supplementation with 200 g/t compound *Bacillus* preparation or 10% fermented soybean hull showed no growth-promoting effects. Neither method adversely affected the measured growth performance, nutrient digestibility, or serum parameters. The present study demonstrates that appropriate doses of Ningxiang pig-derived compound *Bacillus* preparation or its fermented soybean hull can increase final body weight and nutrient digestibility in growing–finishing pigs. These findings provide experimental support for the application of *Bacillus* spp. in feed fermentation and finishing pig production.

## Simple Summary

Soybean hull is an inexpensive by-product of soybean processing, but its relatively high fiber content limits its use in pig diets. We tested two *Bacillus* strains obtained from Ningxiang pigs, *Bacillus subtilis* and *Bacillus licheniformis*, as a means of improving soybean hull and their use in growing–finishing pigs. Both strains showed cellulase-producing activity. When used together for fermentation, they reduced the crude fiber content of soybean hull and increased its crude protein and ether extract concentrations. In the feeding trials, pigs receiving 400 g/t of the compound *Bacillus* preparation or 5% fermented soybean hull had a higher final body weight and greater crude protein digestibility. The compound *Bacillus* preparation also affected several serum antioxidant and inflammatory indicators, whereas fermented soybean hull had fewer effects on these measures and mainly improved protein utilization. Increasing the fermented soybean hull level to 10% did not further improve pig performance. These results provide a possible approach for using Ningxiang pig-derived *Bacillus* strains in feed supplementation and the processing of fibrous feed ingredients.

## 1. Introduction

With the expansion of intensive pig production, the demand for feed ingredients continues to rise, placing increasing pressure on the supply of high-quality feed resources. Protein-rich ingredients such as soybean meal are still heavily reliant on imports, which increases feed costs and may compromise the long-term sustainability of the swine industry [1,2]. Consequently, increasing attention has been paid to the development and utilization of locally available, low-cost feed resources [3,4]. Soybean hull, an abundant by-product of soybean processing, is widely available and relatively inexpensive, making it a potential unconventional feed ingredient for pigs. Its practical use in finishing pig diets, however, is limited by its high crude fiber content and relatively low concentration of readily digestible nutrients, which reduce nutrient utilization and feeding efficiency [5,6]. Improving the nutritional value of soybean hull through appropriate processing strategies could therefore provide a feasible approach for expanding alternative feed resources and reducing the reliance on conventional feed ingredients.

Microbial fermentation is considered an effective strategy for improving the nutritional characteristics of fibrous feed materials. During fermentation, microorganisms and their extracellular enzymes can break down structural carbohydrates, modify the composition of nutrients, and generate additional small-molecule metabolites, thereby improving the availability of nutrients in the fermented substrate [7]. Among the microorganisms used for feed fermentation, *Bacillus* spp. are particularly attractive because of their strong environmental tolerance, relatively simple culture requirements, and ability to produce a broad range of extracellular enzymes, including cellulases and proteases [8,9]. Previous studies have shown that *Bacillus subtilis* (*B. subtilis*) and *Bacillus licheniformis* (*B. licheniformis*) can reduce anti-nutritional components and improve protein utilization, with beneficial effects on intestinal health and growth performance in pigs [10,11]. Nevertheless, many existing studies have focused on commercially available strains. Their application may be constrained by differences in host adaptation, intestinal persistence, and functional performance under specific production conditions [12,13,14]. Strains originating from the target host may offer an alternative, as their adaptation to the intestinal environment of local pig breeds could favor both functional stability and application in feed-related interventions [15,16].

Ningxiang pigs are an important indigenous pig breed in China and possess distinctive genetic and intestinal microbial characteristics, making them a potentially valuable source of functional microorganisms. Several beneficial microorganisms isolated from Ningxiang pigs have already shown promising effects in pig production. For example, *Lactobacillus reuteri* XY227 isolated from the intestine of Ningxiang pigs improved final body weight and average daily gain in Duroc × Landrace × Yorkshire pigs [17]. *Enterococcus hirae* was reported to alleviate Escherichia coli-induced enteritis through its metabolites [18], whereas *Bifidobacterium pseudocatenulatum* reduced excessive fat deposition by promoting secondary bile acid biosynthesis [19]. These findings suggest that the intestinal microbiota of Ningxiang pigs represents a useful reservoir for identifying strains with potential nutritional and health-promoting functions. However, whether Ningxiang pig-derived *Bacillus* spp. can be exploited for the fermentation of unconventional feed ingredients and subsequently applied in finishing-pig production remains largely unexplored.

In the present study, two *Bacillus* strains, *B. subtilis* and *B. licheniformis*, previously isolated and preserved from the fresh feces of Ningxiang pigs in our laboratory, were selected for further evaluation. We first characterized their cellulase-producing capacity and assessed their ability to improve the nutritional characteristics of soybean hull through in vitro fermentation. Based on these findings, two feeding experiments were subsequently conducted to compare the effects of dietary supplementation with a compound *Bacillus* preparation and with soybean hull fermented by the compound *Bacillus* preparation. Growth performance, apparent nutrient digestibility, serum biochemical characteristics, antioxidant status, and immune and inflammatory responses were evaluated in growing–finishing pigs. This study was designed to assess the potential of Ningxiang pig-derived *Bacillus* spp. as feed-associated probiotics and to determine whether their application in soybean hull fermentation could improve the utilization value of this unconventional feed resource.

## 2. Materials and Methods

### 2.1. Ethics Statement

All experimental protocols were reviewed and approved by the Protocol Management and Review Committee at the Institute of Subtropical Agriculture, Chinese Academy of Sciences (protocol code: 20250509; date of approval: 9 May 2025).

### 2.2. Determination of Cellulase Activity Produced by Bacillus spp.

*B. subtilis* and *B. licheniformis* were isolated from the fresh feces of Ningxiang pigs. *Bacillus amyloliquefaciens* (*B. amyloliquefaciens*) ATCC 23350 (American Type Culture Collection, Manassas, VA, USA), a well-characterized cellulase-producing strain, was used as a positive control. Seed cultures of the three strains were enumerated and adjusted to 1.0 × 10^8^ CFU/mL before inoculation into a cellulase production medium containing (g/L): CMC-Na, 10.0; peptone, 5.0; yeast extract, 5.0; KH_2_PO_4_, 1.0; MgSO_4_·7H_2_O, 0.2; and NaCl, 0.5 (pH 7.0) at an inoculation rate of 1% (*v*/*v*). All chemicals were obtained from Sigma-Aldrich (St. Louis, MO, USA). The cultures were incubated in a shaking incubator (Shanghai Zhicheng Analytical Instrument Manufacturing Co., Ltd., Shanghai, China) at 37 °C with shaking at 180 rpm. After 4, 7, and 10 h of incubation, well-mixed fermentation broth samples were collected. Each sample was divided into two portions. The first portion was used for viable bacterial enumeration. The second portion was centrifuged at 10,000× *g* for 15 min at 4 °C in a high-speed centrifuge (Eppendorf, Hamburg, Germany), and the resulting supernatants were collected as crude enzyme extracts. Endo-β-1,4-glucanase (cellulase) activity was determined using a commercial Endo-β-1,4-Glucanase/Cellulase Activity Assay Kit (Boyan, Nanjing, China) according to the manufacturer’s instructions. One unit (U) of cellulase activity was defined as the amount of enzyme required to release 1 μg of reducing sugars per milliliter of enzyme solution per hour under the assay conditions. Measured cellulase activity (U/mL supernatant) was normalized against the corresponding viable cell density (CFU/mL) obtained at each sampling time point and expressed as cellulase activity (U per 1.0 × 10^8^ CFU). The normalization equation was: cellulase activity (U per 1.0 × 10^8^ CFU) = (U/mL ÷ CFU/mL) × 1.0 × 10^8^. Spread-plate viable counting procedure: Bacterial samples were serially 10-fold diluted with sterile 0.85% (*w*/*v*) normal saline. Aliquots of 100 μL from appropriate dilutions were spread uniformly onto LB agar plates (Oxoid, Basingstoke, UK). Plates were incubated aerobically at 37 °C for 24 h. Plates containing 30–300 visible colonies were selected for enumeration. Viable counts were calculated as CFU/mL = (colony number × dilution factor)/volume of inoculum plated (mL).

### 2.3. Fermentation of Soybean Hull

Soybean hull were ground and passed through a 40-mesh sieve to obtain a uniform powder. The fermentation substrate was prepared in 250 mL Erlenmeyer flasks by thoroughly mixing soybean hull powder, soybean meal powder, and distilled water at a ratio of 3:1:1 (*w*/*w*/*w*), with a total substrate weight of 100 g per flask. The prepared substrates were sterilized by autoclaving (HIRAYAMA Manufacturing Corporation, Tokyo, Japan) at 121 °C for 15 min before inoculation. *B. subtilis*, *B. licheniformis*, and *B. amyloliquefaciens* ATCC 23350 were cultured separately in LB broth (Oxoid, Basingstoke, UK) at 37 °C with shaking at 180 rpm for 7 h. Viable plate counting was performed as described in Section 2.2, and the cultures were separately adjusted to 1.0 × 10^8^ CFU/mL with sterile LB broth. For single-strain fermentation, 5 mL of the adjusted *B. subtilis*, *B. licheniformis*, or *B. amyloliquefaciens* suspension was inoculated into sterilized fermentation substrates. For combined fermentation, the adjusted *B. subtilis* and *B. licheniformis* cultures were mixed at a 1:1 (*v*/*v*) ratio, and 5 mL of the mixture was inoculated into sterilized fermentation substrates. For the negative control, 5 mL of sterile LB broth was added instead of the bacterial suspension.

All samples were subjected to solid-state fermentation at 37 °C for 48 h under static conditions. Fermentation was terminated by heating the samples at 105 °C for 20 min. The fermented products were then vacuum freeze-dried (Labconco Corporation, Kansas City, MO, USA) for 72 h, ground, and passed through a 40-mesh sieve for subsequent nutrient analysis. The contents of crude protein (CP), ether extract (EE), crude fiber (CF), and gross energy (GE) in soybean hull substrates before and after fermentation were determined according to the methods of AOAC (2005) [20].

### 2.4. Animal Experiment Design

Experiment 1: A total of 162 healthy growing–finishing pigs (initial body weight of approximately 85 kg, Duroc × Landrace × Yorkshire) were randomly allocated to three dietary treatments, with six replicate pens per treatment and nine pigs per pen. The dietary treatments consisted of: (1) Control (CONT), fed the basal diet; (2) BSL200, fed the basal diet supplemented with 200 g/t of a *B. subtilis* and *B. licheniformis* preparation (10^9^ CFU/g for each bacterium; total: 2 × 10^9^ CFU/g); and (3) BSL400, fed the basal diet supplemented with 400 g/t of the same preparation. The experiment lasted 61 days. The basal diet was a corn–soybean meal-based diet formulated to meet or exceed the nutrient requirements for growing–finishing pigs recommended by the National Research Council (NRC, 2012) [21]. The ingredient composition and nutrient levels of the experimental diets are shown in Supplementary Table S1.

Experiment 2: A total of 126 healthy growing–finishing pigs (Duroc × Landrace × Yorkshire) with an initial body weight of approximately 77 kg were randomly allocated to three dietary treatments, with six replicate pens per treatment and seven pigs per pen. The dietary treatments were as follows: (1) Control (CON), fed a basal diet; (2) FP5, fed the basal diet supplemented with 5% probiotic-fermented soybean hull; and (3) FP10, fed the basal diet supplemented with 10% probiotic-fermented soybean hull. The experiment lasted 60 days. The probiotic-fermented soybean hull was produced by fermentation with a compound probiotic culture (*B. subtilis* and *B. licheniformis*). The ingredient composition and nutrient levels of the experimental diets are presented in Supplementary Table S2.

Before the start of the trials, the pig house was thoroughly disinfected, and all feeders and drinkers were cleaned. All pigs underwent routine vaccination and deworming according to the farm’s standard management procedures. Pigs were fed twice daily at 10:00 and 16:00, with feed offered ad libitum to ensure a small amount of residual feed remained in the feeders after each meal. All animals were housed under identical environmental and management conditions. The ambient temperature was maintained at approximately 25 °C, and the pens were kept clean, dry, well ventilated, and hygienic throughout the experiment. Routine disinfection was carried out according to standard farm procedures. The pigs were monitored daily for general health status, behavior, feed intake, and water consumption, and any abnormalities were promptly recorded and managed. No antibiotics or other probiotic products were administered during the experimental period.

### 2.5. Growth Performance

Body weight was recorded for pigs in each pen after an overnight fast at the beginning and end of the experimental period. Feed intake was recorded for each pen throughout the trial. Initial body weight (IBW) and final body weight (FBW) were recorded, and average daily gain (ADG), average daily feed intake (ADFI), and the feed-to-gain ratio (F/G) were subsequently calculated on a pen basis.

### 2.6. Determination of Serum Biochemical Parameters, Immune and Inflammatory Cytokines, and Antioxidant Indices

At the end of the trial, one pig per pen was selected, and blood samples were collected from the jugular vein of one randomly selected pig per pen following an overnight fast. Serum was obtained by centrifugation (3000× *g*, 15 min, 4 °C) and stored at −80 °C in an ultra-low-temperature freezer (Thermo Fisher Scientific, Waltham, MA, USA) until analysis. Serum biochemical parameters were analyzed using an automatic biochemical analyzer (Hitachi 7020, Tokyo, Japan). Serum immunoglobulins, inflammatory cytokines, and antioxidant indices were quantified using commercial ELISA kits (Boyan, Nanjing, China) according to the manufacturer’s instructions.

### 2.7. Apparent Nutrient Digestibility

Representative feed samples were collected using the quartering method, and approximately 150 g of each sample was placed into labeled sample bags and stored at −20 °C in a freezer (Haier Biomedical, Qingdao, China) until nutrient analysis. During the final 3 days of the experiment, fresh fecal samples were collected from each pen shortly after defecation. Fecal samples collected over the 3-day period were pooled by pen, dried in an oven (Memmert, Schwabach, Germany), and analyzed for crude protein (CP), ether extract (EE), and gross energy (GE). Acid-insoluble ash (AIA) was used as an internal marker to determine the apparent total tract digestibility (ATTD) of nutrients [22].

### 2.8. Statistical Analysis

All data were analyzed by one-way ANOVA using SPSS version 26.0 (IBM Corp., Armonk, NY, USA). Prior to ANOVA, data were tested for normality using the Shapiro–Wilk test and for homogeneity of variances using Levene’s test. Means were compared using Tukey’s honestly significant difference (HSD) test at *p* < 0.05. Results are expressed as mean ± SEM. For growth performance, apparent nutrient digestibility, and other pen-level measurements, the pen was considered the experimental unit. For serum biochemical, immune, inflammatory, and antioxidant indices, one pig per pen was selected for blood sampling, and the resulting observation was treated as an independent biological replicate at the pen level.

## 3. Results

### 3.1. Comparison of Endo-β-1,4-Glucanase/Cellulase Among Bacillus spp. Isolated from Ningxiang Pigs

As shown in Table 1, endo-β-1,4-glucanase/cellulase activity was analyzed separately at each incubation time using one-way ANOVA, and the results showed that cellulase activity of the three *Bacillus* spp. peaked at 4 h and declined thereafter at 7 and 10 h. At 4 h, *B. licheniformis* and *B. amyloliquefaciens* exhibited higher cellulase activity than *B. subtilis*. In contrast, at 7 h, cellulase activity of *B. amyloliquefaciens* was lower than that of *B. subtilis* and *B. licheniformis*. By 10 h, no significant differences in cellulase activity were detected among the three strains.

### 3.2. Nutritional Composition of Soybean Hull After Fermentation with Bacillus spp.

Soybean hulls were fermented with different *Bacillus* spp., and their nutritional composition was analyzed (Table 2). Fermentation with *B. amyloliquefaciens* alone or with a combination of *B. licheniformis* and *B. subtilis* significantly increased crude protein content. Ether extract content was also significantly increased by fermentation with *B. licheniformis*, *B. subtilis*, or both strains combined. All fermentation treatments reduced crude fiber content, with the lowest values observed in soybean hulls fermented with *B. licheniformis* alone or together with *B. subtilis*. Gross energy did not differ among the treatments.

### 3.3. Effects of Dietary Bacillus spp. on Growing–Finishing Pigs

We first evaluated the effects of dietary supplementation with a combination of *B. licheniformis* and *B. subtilis* on the growth performance of growing–finishing pigs. As shown in Table 3, dietary supplementation with the *Bacillus* combination increased FBW, whereas the increase at the low supplementation level was not statistically significant. Dietary supplementation with the *Bacillus* combination had no significant effects on ADG, ADFI, or F/G.

Serum biochemical parameters, immunoglobulins, inflammatory cytokines, and antioxidant indices were subsequently determined in growing–finishing pigs. As shown in Supplementary Table S3, dietary supplementation with the *Bacillus* combination had no significant effects on the activities of aspartate aminotransferase (AST), alanine aminotransferase (ALT), or alkaline phosphatase (ALP), or on the serum concentrations of immunoglobulin A (IgA), immunoglobulin M (IgM), and immunoglobulin G (IgG). As shown in Table 4, dietary supplementation with the *Bacillus* combination significantly increased the activity of glutathione peroxidase (GPX) and superoxide dismutase (SOD), as well as the concentration of anti-inflammatory cytokine interleukin-10 (IL-10), while significantly decreasing the concentration of pro-inflammatory cytokine tumor necrosis factor-α (TNF-α).

### 3.4. Effects of Dietary Bacillus spp. on Apparent Nutrient Digestibility of Growing–Finishing Pigs

We next evaluated the effects of dietary supplementation with the *Bacillus* combination on the apparent nutrient digestibility of growing–finishing pigs. As shown in Table 5, dietary supplementation with the *Bacillus* combination significantly improved the apparent digestibility of CP and GE, whereas the apparent digestibility of EE was not affected.

### 3.5. Effects of Dietary Soybean Hull Fermented by Bacillus spp. on Growth Performance of Growing–Finishing Pigs

We next evaluated the effects of dietary supplementation with *Bacillus*-fermented soybean hulls on growing–finishing pigs. As shown in Table 6, dietary supplementation with the lower, but not the higher, level of *Bacillus*-fermented soybean hulls significantly increased FBW compared with the control group. Dietary supplementation with the lower level also increased ADG and ADFI, although these increases were not statistically significant. Dietary supplementation with *Bacillus*-fermented soybean hulls reduced F/G, but the reduction did not reach statistical significance.

Serum biochemical parameters, immunoglobulins, inflammatory cytokines, and antioxidant indices were subsequently determined in growing–finishing pigs. As shown in Supplementary Table S4, dietary supplementation with *Bacillus*-fermented soybean hulls had no significant effects on the activities of AST, ALT, or ALP, or on the serum concentrations of IgA, IgM, and IgG. As shown in Table 7, dietary supplementation with *Bacillus*-fermented soybean hulls had no significant effects on the activity of GPX or SOD, nor on the concentration of IL-10 of TNF-α.

### 3.6. Effects of Dietary Soybean Hull Fermented by Bacillus spp. on Apparent Nutrient Digestibility of Growing–Finishing Pigs

We next evaluated the effects of dietary supplementation with *Bacillus*-fermented soybean hulls on the apparent nutrient digestibility of growing–finishing pigs. As shown in Table 8, dietary supplementation with *Bacillus*-fermented soybean hulls significantly increased the apparent digestibility of CP, whereas no significant effects were observed on the apparent digestibility of EE or GE.

## 4. Discussion

### 4.1. Cellulase Production by Bacillus spp. and Fermentation Improvement of Soybean Hull

*Bacillus* spp. are widely used as probiotics in pig production because of their diverse effects on both the intestinal environment and feed substrates. In pigs, *Bacillus* supplementation has been associated with improved intestinal health and growth performance, potentially through modulation of the gut microbiota, maintenance of intestinal barrier integrity, and regulation of immune responses [23]. Their ability to produce extracellular enzymes also makes them suitable for the bioconversion of fibrous feed materials, particularly those containing substantial amounts of cellulose [24]. In the present study, we first evaluated the cellulase-producing ability of two *Bacillus* strains isolated from Ningxiang pigs. The two strains showed distinct enzyme production patterns during fermentation. At 4 h, cellulase activity of *B. licheniformis* was comparable to that of *B. amyloliquefaciens*, and both were higher than that of *B. subtilis*. By 7 h, however, *B. subtilis* and *B. licheniformis* exhibited higher cellulase activities than *B. amyloliquefaciens*. These time-dependent differences suggest that the strains may differ in their enzyme production dynamics. Importantly, the relatively high cellulase-producing capacity of *B. subtilis* and *B. licheniformis* supports their potential application in the bioconversion of high-fiber feed ingredients.

Soybean hull is an abundant by-product of soybean processing, but its relatively high fiber content restricts its inclusion in diets for growing–finishing pigs. Solid-state fermentation provides a practical means of modifying the nutrient profile of fibrous feed ingredients by allowing microorganisms and their enzymes to utilize and transform complex substrates [25]. We therefore further examined whether the selected *Bacillus* strains could improve the nutritional characteristics of soybean hull. Fermentation with either strain increased the measured CP and EE concentrations while reducing CF concentrations, although the extent of these changes differed between strains. *B. subtilis* showed a greater effect on CP and EE, whereas *B. licheniformis* exhibited a stronger capacity to reduce CF. The complementary characteristics of the two strains may explain the more pronounced changes observed following co-fermentation. Indeed, combined fermentation with *B. subtilis* and *B. licheniformis* reduced CF from 24.99% to 6.57%, while CP and EE increased from 19.47% and 5.63% to 22.91% and 7.89%, respectively. These changes indicate that co-fermentation produced a more favorable overall nutrient profile than fermentation with either strain alone.

The increase in CP following fermentation may partly reflect the contribution of microbial biomass and the redistribution of nutrients during substrate degradation rather than a simple net accumulation of protein. Likewise, the apparent increase in EE may be related to changes in the relative composition of the fermented substrate as carbohydrates and other readily utilized components were consumed. The marked reduction in CF, particularly after co-fermentation, is consistent with the cellulase-producing capacity observed in the strain screening experiment and suggests that the two strains may act complementarily during the degradation of fibrous components. Similar findings have been reported for fermented soybean meal. Dai et al. found that solid-state fermentation with *B. licheniformis* and *B. subtilis* increased CP concentration, although the magnitude of the response varied among strains [26]. Zhao et al. further showed that co-fermentation with these two *Bacillus* strains increased soluble protein and reduced anti-nutritional factors in soybean meal [27]. Together with our findings, these studies support the feasibility of using *Bacillus*-based fermentation to improve the nutritional characteristics of plant-derived feed by-products. Interestingly, GE did not differ significantly among the fermentation treatments, despite the substantial changes in CF, CP, and EE. This suggests that fermentation did not cause a measurable loss of gross energy at the substrate level, while simultaneously altering its nutrient composition. However, GE represents the total energy released by combustion and does not directly reflect the amount of energy available to the animal. Therefore, the improved nutritional profile observed after fermentation needs to be further evaluated through feeding trials and measurements of nutrient digestibility. The subsequent animal experiments were thus conducted to determine whether the compositional changes induced by *Bacillus* fermentation could translate into improved nutrient utilization and physiological responses in growing–finishing pigs.

To assess whether the two Ningxiang pig-derived *Bacillus* spp. could exert beneficial effects through different feeding strategies, we conducted two feeding trials in growing–finishing pigs using either a compound *Bacillus* preparation or soybean hull fermented with the same bacterial combination.

### 4.2. Effects of the Compound Bacillus Preparation on Growing–Finishing Pigs

The results showed that supplementation with 200 g/t of the compound *Bacillus* preparation did not significantly affect growth performance, whereas increasing the supplementation level to 400 g/t resulted in a significant increase in FBW. A similar has been reported by Alexopoulos et al., who observed greater growth-promoting effects at medium and high supplementation levels when a combined spore preparation of *B. subtilis* and *B. licheniformis* was included in finishing pig diets [28]. The different responses to the two supplementation levels in the present study suggest that the beneficial effects of the compound preparation may depend, at least in part, on the amount of viable bacteria supplied.

The improvement in body weight was accompanied by changes in nutrient utilization. Dietary supplementation with the compound *Bacillus* preparation significantly increased the apparent digestibility of CP and GE, suggesting that the bacterial preparation may have improved the utilization of dietary protein and energy. Similar improvements in nutrient digestibility following *Bacillus* supplementation have been reported previously [29]. However, the increase in nutrient digestibility at the lower supplementation level was not accompanied by a detectable improvement in FBW. This discrepancy suggests that changes in nutrient utilization do not necessarily translate directly into measurable differences in body weight over the experimental period. The greater FBW observed at 400 g/t may be related to the improved nutrient utilization observed at this supplementation level, although other factors may also have contributed. Further studies involving longer feeding periods and measurements of intestinal microbial and metabolic responses would be useful for clarifying the mode of action of the compound preparation.

Immune, antioxidant, and serum biochemical measurements provide useful information on the physiological status of growing–finishing pigs. In the present study, supplementation with the compound *Bacillus* preparation did not significantly affect serum IgA, IgG, or IgM concentrations, nor did it alter the activities of AST, ALT, or ALP (Table S3). Similar results have been reported in pigs receiving *Bacillus*-based preparations [30,31]. The absence of detectable changes in these parameters, together with the improvements observed in FBW and nutrient digestibility, suggests that the tested supplementation levels did not impose an apparent adverse effect on the measured serum immune parameters or liver enzyme activities. More importantly, the compound *Bacillus* preparation increased serum SOD and GPX activities, accompanied by an increase in IL-10 and a reduction in TNF-α. These changes indicate an improved systemic antioxidant status and a shift toward a less inflammatory serum profile. The antioxidant effects may be related to the ability of *Bacillus* spp. to interact with the intestinal environment and influence host redox homeostasis, although the underlying mechanisms were not directly examined in the present study.

### 4.3. Effects of Fermented Soybean Hull on Growing–Finishing Pigs

In the fermented soybean hull trial, inclusion of 5% fermented soybean hull significantly increased FBW and CP apparent digestibility, whereas ADG, ADFI, F/G, and GE digestibility were not significantly affected. As described above, co-fermentation with *B. subtilis* and *B. licheniformis* reduced CF while increasing the measured CP concentration of soybean hull. Microbial fermentation may also alter the form and accessibility of dietary protein. Dai et al. reported that solid-state fermentation of soybean meal with *B. subtilis* increased its free amino acid content [32]. Similarly, Jang et al. found that fermentation with *B. licheniformis* increased the abundance of serine, threonine, and glutamate, whereas *B. subtilis* promoted the accumulation of lysine, leucine, and tryptophan in fermented soybean substrates [33]. These observations suggest that microbial fermentation can modify protein substrates and their amino acid profiles, potentially facilitating subsequent digestion and absorption. Thus, the increased FBW and CP digestibility observed in pigs receiving 5% fermented Soybean hull may be partly attributable to the improved accessibility and nutritional quality of protein following fermentation. Notably, increasing the inclusion level of fermented soybean hull from 5% to 10% did not result in further improvements in FBW or apparent nutrient digestibility. One possible explanation is that the additional benefits provided by fermentation were insufficient to compensate for the greater fiber load at the higher inclusion level. Although fermentation substantially reduced the CF concentration of soybean hull, the 10% inclusion level would still provide more fiber to the diet than the 5% level. An increased supply of indigestible or slowly fermentable fiber may limit the extent to which the improved nutritional characteristics of fermented soybean hull can be translated into enhanced nutrient utilization. However, this interpretation remains speculative because the present study did not directly assess fiber fermentation, gastrointestinal transit, or microbial utilization of the additional fiber. Therefore, the lack of a further response at the 10% inclusion level warrants additional investigation.

In addition, fermented soybean hull did not cause significant changes in serum immunoglobulins, antioxidant enzymes, or inflammatory cytokines in growing–finishing pigs. This may be partly due to the subsequent drying of the fermented soybean hull, which may have reduced the viability of the bacterial strains; however, viable bacterial counts after drying were not determined in this study. Therefore, this explanation remains speculative. Furthermore, the absence of significant systemic immune and antioxidant responses in the fermented soybean hull groups may also be related to factors such as bacterial dose, fermentation-derived metabolites, and sampling time and should not be attributed solely to bacterial viability. Overall, the two *Bacillus* application strategies had different effects on serum immune and antioxidant indices, and their relationships with intestinal local immunity, digestive enzyme activity, and hindgut microbial fermentation still require further investigation.

### 4.4. Potential Mechanisms and Limitations

The present study did not directly assess fiber fermentation, gastrointestinal transit, or microbial utilization of the additional fiber, and the mechanisms by which the two *Bacillus* feeding strategies affect growth performance in growing–finishing pigs remain speculative. Future studies should focus on measuring the activities of protein digestion-related enzymes in the stomach and small intestine to verify whether *Bacillus* or fermented soybean hull promotes protein and fiber digestion by enhancing host digestive enzyme activity. In addition, the structure and metabolites of the hindgut microbiota should be further determined to evaluate the contribution of changes in gut microbial fermentation to the growth-promoting effects.

## 5. Conclusions

In conclusion, co-fermentation with Ningxiang pig-derived *B. subtilis* and *B. licheniformis* improved the nutritional characteristics of soybean hull, as evidenced by reduced CF and increased CP and EE concentrations. Dietary supplementation with 400 g/t compound *Bacillus* preparation or 5% fermented soybean hull increased FBW and CP apparent digestibility in growing–finishing pigs, while direct *Bacillus* supplementation additionally enhanced serum antioxidant capacity and favorably modulated inflammatory status. These findings support the potential application of Ningxiang pig-derived *Bacillus* spp. as feed probiotics and as biological agents for the improvement of the nutrient composition and utilization of unconventional fibrous feed resources.

## Figures and Tables

**Table 1:** Cellulase activity produced by *Bacillus* spp. at different time points.

Enzyme Activity, U per 1.0 × 10^8^ CFU	BA	BS	BL
4 h	35.6 ± 1.4 ^a^	32.7 ± 1.2 ^b^	35.9 ± 4.0 ^a^
7 h	23.1 ± 0.5 ^b^	25.8 ± 0.7 ^a^	25.1 ± 1.4 ^a^
10 h	24.7 ± 1.4	26.1 ± 1.0	25.9 ± 2.2

^a,b^ Different superscript letters in the same row indicate significant differences (*p* < 0.05). Data are expressed as Mean ± SEM. *n* = 6. BA, *Bacillus amyloliquefaciens*; BS, *Bacillus subtilis*; BL, *Bacillus licheniformis*.

**Table 2:** The nutritional composition of soybean hull after fermentation with *Bacillus* spp.

	CONT	BA	BS	BL	BSL
CP, %	19.47 ± 0.04 ^c^	22.43 ± 1.43 ^ab^	21.62 ± 0.46 ^bc^	20.22 ± 0.29 ^c^	22.91 ± 0.08 ^a^
EE, %	5.63 ± 0.18 ^c^	5.66 ± 0.43 ^c^	10.55 ± 1.54 ^a^	8.51 ± 1.43 ^b^	7.89 ± 0.28 ^b^
CF, %	24.99 ± 3.14 ^a^	13.32 ± 0.16 ^b^	13.57 ± 1.65 ^b^	7.84 ± 0.87 ^c^	6.57 ± 1.83 ^c^
GE, MJ/kg	18.97 ± 1.35	18.77 ± 0.39	18.99 ± 0.19	18.96 ± 0.93	18.73 ± 0.82

^a,b,c^ Different superscript letters in the same row indicate significant differences (*p* < 0.05). Data are expressed as Mean ± SEM. *n* = 3. CONT, unfermented soybean hull; BA, soybean hull fermented with *Bacillus amyloliquefaciens*; BS, soybean hull fermented with *Bacillus subtilis*; BL, soybean hull fermented with *Bacillus licheniformis*; BSL, soybean hull co-fermented with *Bacillus subtilis* and *Bacillus licheniformis*. CP, crude protein; EE, ether extract; CF, crude fiber; GE, gross energy.

**Table 3:** Effects of dietary *Bacillus* spp. on growth performance of growing–finishing pigs.

	CONT	BSL200	BSL400
IBW, kg	84.5 ± 1.7	85.2 ± 5.1	84.9 ± 2.3
FBW, kg	150.9 ± 2.7 ^b^	158.7 ± 9.7 ^ab^	158.8 ± 2.4 ^a^
ADG, kg/d	1.08 ± 0.06	1.14 ± 0.04	1.22 ± 0.04
ADFI, kg/d	3.87 ± 0.08	3.87 ± 0.23	3.92 ± 0.15
F/G	3.58 ± 0.14	3.40 ± 0.28	3.22 ± 0.21

^a,b^ Different superscript letters in the same row indicate significant differences (*p* < 0.05). Data are expressed as Mean ± SEM. *n* = 6. CONT, basal diet; BSL200, basal diet supplemented with 200 g/t compound *Bacillus* preparation; BSL400, basal diet supplemented with 400 g/t compound *Bacillus* preparation. IBW, initial body weight; FBW, final body weight; ADG, average daily gain; ADFI, average daily feed intake; F/G, feed-to-gain ratio.

**Table 4:** Effects of dietary *Bacillus* spp. on inflammatory cytokines and antioxidant indices of growing–finishing pigs.

	CONT	BSL200	BSL400
GPX, nmol/min/mL	599 ± 13 ^b^	616 ± 10 ^a^	617 ± 15 ^a^
SOD, U/mL	44.8 ± 2.8 ^b^	50.0 ± 5.5 ^ab^	53.7 ± 9.4 ^a^
IL-10, pg/mL	34.4 ± 2.7 ^b^	42.2 ± 4.6 ^a^	45.0 ± 2.2 ^a^
TNF-α, pg/mL	55.2 ± 6.0 ^a^	40.9 ± 3.0 ^b^	39.6 ± 3.0 ^b^

^a,b^ Different superscript letters in the same row indicate significant differences (*p* < 0.05). Data are expressed as Mean ± SEM. *n* = 6. CONT, basal diet; BSL200, basal diet supplemented with 200 g/t compound *Bacillus* preparation; BSL400, basal diet supplemented with 400 g/t compound *Bacillus* preparation. GPX, glutathione peroxidase; SOD, superoxide dismutase; IL-10, interleukin-10; TNF-α, tumor necrosis factor-α.

**Table 5:** Effects of dietary *Bacillus* spp. on apparent nutrient digestibility of growing–finishing pigs.

	CONT	BSL200	BSL400
CP, %	73.3 ± 1.8 ^b^	76.5 ± 2.3 ^a^	76.6 ± 1.6 ^a^
EE, %	82.7 ± 1.5	82.1 ± 0.7	83.2 ± 2.7
GE, %	79.2 ± 1.8 ^c^	82.7 ± 1.0 ^b^	84.4 ± 1.1 ^a^

^a,b,c^ Different superscript letters in the same row indicate significant differences (*p* < 0.05). Data are expressed as Mean ± SEM. *n* = 6. CONT, basal diet; BSL200, basal diet supplemented with 200 g/t compound *Bacillus* preparation; BSL400, basal diet supplemented with 400 g/t compound *Bacillus* preparation. CP, crude protein; EE, ether extract; GE, gross energy.

**Table 6:** Effects of dietary soybean hull fermented by *Bacillus* spp. on growth performance of growing–finishing pigs.

	CON	FP5	FP10
IBW, kg	77.4 ± 3.0	77.6 ± 2.3	77.2 ± 2.0
FBW, kg	123.2 ± 2.7 ^b^	129.0 ± 4.8 ^a^	123.8 ± 4.9 ^ab^
ADG, kg/d	0.77 ± 0.02	0.85 ± 0.11	0.78 ± 0.10
ADFI, kg/d	2.36 ± 0.08	2.51 ± 0.28	2.32 ± 0.10
F/G	3.10 ± 0.11	2.93 ± 0.16	3.02 ± 0.39

^a,b^ Different superscript letters in the same row indicate significant differences (*p* < 0.05). Data are expressed as Mean ± SEM. *n* = 6. CON, basal diet; FP5, basal diet supplemented with 5% fermented soybean hull; FP10, basal diet supplemented with 10% fermented soybean hull. IBW, initial body weight; FBW, final body weight; ADG, average daily gain; ADFI, average daily feed intake; F/G, feed-to-gain ratio.

**Table 7:** Effects of dietary soybean hull fermented by *Bacillus* spp. on inflammatory cytokines and antioxidant indices of growing–finishing pigs.

	CON	FP5	FP10
GPX, nmol/min/mL	542.2 ± 26.0	559.3 ± 21.0	570.0 ± 28.2
SOD, U/mL	62.3 ± 4.1	57.2 ± 3.8	59.3 ± 4.8
IL-10, pg/mL	22.8 ± 2.1	24.0 ± 2.7	24.1 ± 1.8
TNF-α, pg/mL	41.3 ± 3.3	36.8 ± 4.1	38.6 ± 3.6

Data are expressed as Mean ± SEM. *n* = 6. CON, basal diet; FP5, basal diet supplemented with 5% fermented soybean hull; FP10, basal diet supplemented with 10% fermented soybean hull. GPX, glutathione peroxidase; SOD, superoxide dismutase; IL-10, interleukin-10; TNF-α, tumor necrosis factor-α.

**Table 8:** Effects of dietary soybean hull fermented by *Bacillus* spp. on apparent nutrient digestibility of growing–finishing pigs.

	CON	FP5	FP10
CP, %	77.1 ± 1.22 ^b^	78.6 ± 0.80 ^a^	78.2 ± 1.43 ^ab^
EE, %	85.0 ± 1.44	84.5 ± 0.72	83.9 ± 2.22
GE, %	83.7 ± 0.74	84.6 ± 0.57	84.5 ± 2.32

^a,b^ Different superscript letters in the same row indicate significant differences (*p* < 0.05). Data are expressed as Mean ± SEM. *n* = 6. CON, basal diet; FP5, basal diet supplemented with 5% fermented soybean hull; FP10, basal diet supplemented with 10% fermented soybean hull. CP, crude protein; EE, ether extract; GE, gross energy.

## Data Availability

The data presented in this study are available from the corresponding authors upon reasonable request.

## Supplementary Materials

The following supporting information can be downloaded at: https://www.mdpi.com/article/10.3390/ani16203224/s1, Table S1: Ingredient composition and calculated nutrient levels of the experimental diets in Experiment 1; Table S2: Ingredient composition and calculated nutrient levels of the experimental diets in Experiment 2; Table S3: Effects of dietary *Bacillus* spp. on serum biochemical parameters and immunoglobulins content of growing-finishing pigs; Table S4: Effects of dietary soybean hull fermented by *Bacillus* spp. on serum biochemical parameters and immunoglobulins content of growing-finishing pigs.
