# Supplementary material for: Effects of Dietary Bacillus spp. and Bacillus spp.-Fermented Soybean Hull on Growth Performance and Apparent Nutrient Digestibility in Growing–Finishing Pigs
Source: Animals (Basel). 2026 Oct 14;16(20):3224. doi: 10.3390/ani16203224 (PMC13700783; doi:10.3390/ani16203224)
Supplement: Supplementary file 1 [file animals-16-03224-s001.zip › animals-4587485-supplementary.pdf]

Supplementary Table S1 Ingredient composition and calculated nutrient levels of the experimental diets in Experiment 1

Ingredients	%	Calculated value	
Corn	70.0	DE, MJ/kg	14.14
Soybean meal	17.5	CP, %	13.87
Fat powder	2.5	Ca, %	0.62
Dicalcium phosphate	0.8	Total P, %	0.58
Limestone	1.0	P, %	0.26
Salt	0.5	SID-Lys, %	1.04
Choline chloride 50%	0.1	SID-Met, %	0.25
L-Lys sulfate	1.0	SID-Thr, %	0.41
DL-Methionine	0.05	SID-Trp, %	0.12
Complex vitamin[1]	0.05		
Trace element[2]	0.1		
Sodium bicarbonate	0.2		
Rice bran	6.2		
Total	100		

[1] Provided per kg of diet: vitamin A, 32.5 million IU; vitamin B_1, 10 g; vitamin B_2, 25 g; vitamin B_6, 15 g; vitamin B_{12}, 100 mg; vitamin D_3, 10 million IU; vitamin E, 80 g; vitamin K_3, 10 g; nicotinamide, 120 g; D-pantothenic acid, 70 g; folic acid, 5 g; D-biotin, 500mg.

[2] Provided for per kilogramof diet: $FeSO_4 \cdot H_2O$, 348.97 g; $CuSO_4 \cdot 5H_2O$, 48.83 g; $ZnSO_4 \cdot H_2O$, 193.21 g; $MnSO_4 \cdot H_2O$, 84.51 g; $Ca\ (IO_3)_2 \cdot H_2O$, 0.768 g; $Na_2SeO_3 \cdot 5H_2O$, 1.18 g; Fe, 115 g; Cu, 12.5 g; Zn, 70 g; Mn, 27.5 g; I, 0.5 g; Se, 0.35 g.

Supplementary Table S2 Ingredient composition and calculated nutrient levels of the experimental diets in Experiment 2

Ingredients	0%	5%	10%
Corn	70.00	66.60	66.40
Soybean meal	17.50	15.90	13.10
Fermented soybean hull	0.00	5.00	10.00
Fat powder	2.50	4.50	4.20
Dicalcium phosphate	0.80	0.93	0.95
Limestone	1.00	0.82	0.70
Salt	0.50	0.50	0.50
Choline chloride 50%	0.10	0.10	0.10
L-Lys sulfate	1.00	1.02	1.08
DL-Methionine	0.05	0.06	0.07
L-Threonine	0.00	0.02	0.04
L-Tryptophan	0.00	0.00	0.01
Complex vitamin[1]	0.10	0.10	0.10
Trace element[2]	0.10	0.20	0.20
Sodium bicarbonate	0.20	0.20	0.20
Rice bran	6.20	4.20	2.50
Total	100.00	100.00	100.00
Calculated nutrient levels			
DM, MC/kg	14.14	14.10	14.14
CP, %	13.87	13.87	13.87
Ca, %	0.62	0.62	0.62
Total P, %	0.58	0.51	0.49
P, %	0.26	0.26	0.26
SID-Lys, %	1.04	1.04	1.04
SID-Met, %	0.25	0.25	0.25
SID-Thr, %	0.41	0.41	0.41

SID-Trp, %	0.12	0.12	0.12

[1] Provided per kg of diet: vitamin A, 32.5 million IU; vitamin B_1, 10 g; vitamin B_2, 25 g; vitamin B_6, 15 g; vitamin B_{12}, 100 mg; vitamin D_3, 10 million IU; vitamin E, 80 g; vitamin K_3, 10 g; nicotinamide, 120 g; D-pantothenic acid, 70 g; folic acid, 5 g; D-biotin, 500mg.

[2] Provided for per kilogramof diet: $FeSO_4 \cdot H_2O$, 348.97 g; $CuSO_4 \cdot 5H_2O$, 48.83 g; $ZnSO_4 \cdot H_2O$, 193.21 g; $MnSO_4 \cdot H_2O$, 84.51 g; Ca $(IO_3)_2 \cdot H_2O$, 0.768 g; $Na_2SeO_3 \cdot 5H_2O$, 1.18 g; Fe, 115 g; Cu, 12.5 g; Zn, 70 g; Mn, 27.5 g; I, 0.5 g; Se, 0.35 g.

Supplementary Table S3 Effects of dietary *Bacillus* spp. on serum biochemical parameters and immunoglobulins content of growing-finishing pigs

	CONT	BSL200	BSL400
AST, U/L	22.4±1.5	22.9±2.7	23.2±2.8
ALT, U/L	43.7±1.7	44.4±1.7	44.1±1.8
ALP, U/L	107.8±7.0	112.4±5.6	118.2±15.0
IgA, g/L	1.32±0.07	1.35±0.05	1.42±0.19
IgG, g/L	21.3±3.2	21.1±1.7	22.6±3.5
IgM, g/L	2.24±0.16	2.28±0.23	2.42±0.27

Values are presented as mean ± SEM. n = 6.

Supplementary Table S4 Effects of dietary soybean hull fermented by *Bacillus* spp. on serum biochemical parameters and immunoglobulins content of growing-finishing pigs

	0%	5%	10%
AST，U/L	26.4 ± 2.2	27.9 ± 1.9	29.3 ± 3.5
ALT, U/L	39.5 ± 3.2	36.7 ± 2.8	40.4 ± 4.0
ALP，U/L	96.2 ± 6.4	94.4 ± 7.7	97.9 ± 5.3
IgA，g/L	1.61 ± 0.13	1.57 ± 0.08	1.64 ± 0.11
IgG，g/L	18.4 ± 2.7	19.5 ± 3.2	20.6 ± 2.5
IgM，g/L	2.15 ± 0.12	2.27 ± 0.14	2.19 ± 0.10

Values are presented as mean ± SEM. n = 6.
